# Extraordinary structural complexity of ilmajokite: a multilevel hierarchical framework structure of natural origin

**DOI:** 10.1107/S2052252519016622

**Published:** 2020-01-01

**Authors:** Andrey A. Zolotarev, Sergey V. Krivovichev, Fernando Cámara, Luca Bindi, Elena S. Zhitova, Frank Hawthorne, Elena Sokolova

**Affiliations:** a Department of Crystallography, St Petersburg State University, University Emb. 7/9, St Petersburg 199034, Russian Federation; b Nanomaterials Research Center, Kola Science Center, Russian Academy of Sciences, Fersmana Str. 14, Apatity, Murmansk region 184209, Russian Federation; c Dipartimento di Scienze della Terra, Unversità di Milano, via Mangiagalli 34, Milano I-20133, Italy; d Dipartimento di Scienze della Terra, Università degli Studi di Firenze, Firenze I-50121, Italy; e Laboratory of Mineralogy, Institute of Volcanology and Seismology, Russian Academy of Sciences, Piyp Bulvar 9, Petropavlovsk-Kamchatsky 683006, Russian Federation; f Department of Geological Sciences, University of Manitoba, Winnipeg, Manitoba R3T2N2 Canada

**Keywords:** ilmajokite, titanosilicates, structural hierarchy, structural complexity

## Abstract

The crystal structure of ilmajokite, a rare Na-K-Ba-Ce-titanosilicate from the Khibiny mountains, Kola peninsula, Russia, has been solved using single-crystal X-ray diffraction data. The crystal structure is based on a 3D titanosilicate framework consisting of trigonal prismatic titanosilicate (TPTS) clusters centered by Ce^3+^ in [9]-coordination. Four adjacent TPTS clusters are linked into four-membered rings within the (010) plane and connected *via* ribbons parallel to 101. The ribbons are organized into layers parallel to (010) and modulated along the *a* axis with a modulation wavelength of *c*sinβ = 32.91 Å and an amplitude of ∼*b*/2 = 13.89 Å. The layers are linked by additional silicate tetrahedra. Na^+^, K^+^, Ba^2+^ and H_2_O groups occur in the framework cavities and have different occupancies and coordination environments. The crystal structure of ilmajokite can be separated into eight hierarchical levels: atoms, coordination polyhedra, TPTS clusters, rings, ribbons, layers, the framework and the whole structure. The information-based analysis allows estimation of the complexity of the structure as 8.468 bits per atom and 11990.129 bits per cell. According to this analysis, ilmajokite is the third-most complex mineral known to date after ewingite and morrisonite, and is the most complex mineral framework structure, comparable in complexity to paulingite-(Ca) (11 590.532 bits per cell).

## Abstract 2

Ilmajokite, a natural titanosilicate found in the Russian Arctic, has an extraordinary complex microporous framework structure consisting of several levels of hierarchical organization.

## 1. Introduction   

Minerals constitute a distinct group of crystalline materials formed by natural geochemical or biogeochemical processes without any anthropogenic influence. Approximately 5500 different mineral species are known today with more than 100 new species discovered every year. Many of these minerals have their synthetic counterparts, but there are many minerals that have no artificial analogs (Khomyakov, 1994 ▸). Their existence and formation under natural conditions represent a serious challenge for both mineralogists and material scientists looking for new structural architectures with interesting physical and chemical properties. Recently, a number of highly complex minerals has been characterized with structural features never before seen in synthetic materials. In particular, the crystal structures of charoite and denisovite are based on different kinds of silicate nanotubules (Rozhdestvenskaya *et al.*, 2009 ▸, 2010 ▸, 2017 ▸), and ewingite (Olds *et al.*, 2017 ▸) and morrisonite (Kampf *et al.*, 2016 ▸) contain novel types of nanoscale heteropolyhedral clusters.

One interesting group of minerals with unique and important properties are titanosilicates. Due to their microporosity and catalytic activity, titanosilicates continue to attract considerable attention in the fields of materials science and nanochemistry (Rocha & Anderson, 2000 ▸; Noh *et al.*, 2012 ▸; Milyutin *et al.*, 2017 ▸; Oleksiienko *et al.*, 2017 ▸; Přech, 2018 ▸; Cuko *et al.*, 2018 ▸; Figueiredo *et al.*, 2018 ▸). At the same time, they are of great interest from the viewpoint of mineralogy and geochemistry given their large diversity mostly in alkaline rocks such as those occurring in the alkaline massifs of the Kola peninsula, Russia, and Mont-Saint-Hilaire, Quebec, Canada (Cámara *et al.*, 2017 ▸; Sokolova *et al.*, 2017 ▸; Sokolova & Cámara, 2017 ▸; Selivanova *et al.*, 2018 ▸; Zolotarev *et al.*, 2018 ▸; Andrade *et al.*, 2018 ▸; Lykova *et al.*, 2018 ▸; Pakhomovsky *et al.*, 2018 ▸; Pekov *et al.*, 2019 ▸; Zhitova *et al.*, 2019 ▸). Indeed, most titanosilicate materials used in industry were found as minerals before their unique properties were recognized. For instance, ETS-4 (Engelhardt titanosilicate-4; Kuznicki *et al.*, 2001 ▸) is a synthetic analog of zorite, first described by Soviet mineralogists in the Kola peninsula in 1973 (Mer’kov *et al.*, 1973 ▸). The ion-exchanger UOP-910, which is used for the removal of Cs-137 from radioactive-waste solutions (Anthony *et al.*, 1994 ▸), is a natural counterpart of sitinakite, a microporous titanosilicate from hydrothermal veins of the Khibiny massif, Kola peninsula, Russia (Sokolova *et al.*, 1989 ▸; Men’shikov *et al.*, 1992 ▸). Another family of recently described microporous titanosilicates, the ivanyukite-group minerals (Yakovenchuk *et al.*, 2009 ▸), have the pharmacosiderite-type structure, well known as a useful synthetic material since the 1990s (Harrison *et al.*, 1995 ▸). There are many other unique natural titanosilicates with interesting structures that have no precedents among synthetic materials. For instance, yuksporite is based on nanoscale porous titanosilicate tubes that have never been prepared under laboratory conditions to date (Krivovichev *et al.*, 2004 ▸).

Herein we report on the structural and chemical features of ilmajokite, a rare titanosilicate from the Lovozero tundra, Kola peninsula, Russia. The mineral occurs in the ‘Yubileynaya’ pegmatite vein, Karnasurt Mountain, near the river Ilmajok and lake Ilma. Ilmajokite is found as crystals and crystalline crusts on the surfaces of voids in natrolite (Bussen *et al.*, 1972 ▸). The fresh crystals are yellowish and transparent. In air, they slowly become cloudy and fragment into separate plates and fibers. The chemical formula, determined by wet chemical analysis, was given as (Na_8.8_Ba_0.5_ *REE*_0.7_)_Σ = 10_(Ti_4.99_(Fe,Al,Nb)_0.01_)_Σ = 5_(Si_13.9_Al_0.01_)_Σ = 14_O_22_(OH)_44_·*n*H_2_O (the formula was proposed by I. D. Borneman-Starynkevich). The admixture of nahcolite, NaHCO_3_, was mentioned, which significantly increased the observed amount of Na. Ilmajokite has a large quantity of H_2_O (24.54 wt%), which is released on heating; the mineral loses up to 12.7% at 175°C, although 6.5% is still retained at 320°C. Release of H_2_O starts as low as 60°C but it is not complete until 760°C (Bussen *et al.*, 1972 ▸). H-speciation was determined by infra-red (IR) spectroscopy based on broad absorptions at 1618 and 3389–2889 cm^−1^.

The instability of ilmajokite crystals under atmospheric conditions prevented detailed crystallographic study for a long time. Bussen *et al.* (1972 ▸) determined the mineral to be probably monoclinic with unit-cell parameters *a* ≃ 23, *b* ≃ 24.4, *c* ≃ 37 Å. A later study by Goiko *et al.* (1974 ▸) on fresh material held hermetically after extraction from the pegmatite confirmed the monoclinic symmetry and determined the unit-cell parameters to be *a* = 39.80, *b* = 29.5, *c* = 29.83 Å, β = 96.63°, *V* = 34788 Å^3^, possible space groups *C*2/*c* or *Cc*. Cámara *et al.* (2010 ▸) investigated single crystals of ilmajokite provided by E. I. Semenov in 2004 (sample ILM01). The study at room temperature gave a *C*-centered monoclinic cell with *a* = 35.774 (4), *b* = 27.407 (3), *c* = 31.131 (5) Å, β = 95.66 (1)°, *V* = 30374 (7) Å^3^. The *R*_int_ value was very high (∼32%) and no model could be obtained from these data. A low-temperature data collection at 125 K slightly enhanced the data quality, but no solution could be found. The cell refinement confirmed a *C*-centered cell with *a* = 35.32 (16), *b* = 26.93 (12), *c* = 30.68 (14) Å, β = 95.84 (2)°, *V* = 29034 (403) Å^3^. A further study was done later by some of the authors (FC, LB, FCH and ES) on another crystal provided by C. Ferraris (Muséum National d’Histoire Naturelle, Paris, France; catalog number 197.68) in 2010 (ILM02), extracted from a sample deposited at the National School of Mines, Paris, France. This time, a high-performing BRUKER APEX II ULTRA single-crystal diffractometer was used (Turbo X-ray source coupled with the HELIOS Mo optics provides up to 60 times more intense data from small crystals) at the Department of Geological Sciences of the University of Manitoba, Canada. The results were very similar to the previous ones [refined unit cell: *a* = 36.084 (18), *b* = 27.726 (13), *c =* 31.248 (15) Å, β = 98.051 (5)°, *V* = 30953 (45) Å^3^] but with a better internal agreement factor (*R*_int_ ≃ 5%). Yet again, systematic absences were compatible with space groups *C*2/*c* or *Cc*, but no model could be obtained from these data as they were very weak at *d* < 2.3 Å (Fig. S1 of the supporting information), and show high mosaicity. Interestingly, mosaicity begins at lower resolution along 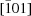 [Fig. S1(*b*)].

Considerable attempts have been made by separate groups to obtain the structure model for ilmajokite, but with no success. Recently, the first author (AAZ) found a single crystal of ilmajokite in the collections of the Fersman Mineralogical Museum of the Russian Academy of Sciences (Moscow, Russia) which allowed data collection good enough to resolve the atomic arrangement of this unusual mineral. The good quality of the crystal was due to the fact that it was covered by lacquer soon after extraction of the sample from the host rock.

## 2. Materials and methods   

### 2.1. Sample description   

The holotype sample was obtained from the Fersman Mineralogical Museum of the Russian Academy of Sciences (Moscow, Russia), where it is stored under catalog number 86969. The sample originates from the Yubileinaya pegmatite (Karnasurt Mountain, Lovozero, Kola Peninsula) (Pekov, 2001 ▸) where it is one of the latest primary minerals. We checked several single crystals and we were able to obtain acceptable X-ray diffraction data for one of them. The crystal selected for data collection was a yellowish transparent plate was 0.15 × 0.08 × 0.04 mm in size.

### 2.2. Chemical composition   

The chemical composition of ilmajokite was determined on ilmajokite crystals from the sample ILM02 using a CAMECA SX-100 electron-microprobe at the Department of Geological Sciences of the University of Manitoba, Canada, operating in wavelength-dispersion mode with an accelerating voltage of 15 kV, a specimen current of 5 nA, a beam size of 15 µm and count times on peak and background of 20 and 10 s, respectively. The results are reported in Table S1 of the supporting information. Even with such a large beam, the material showed significant beam damage (Fig. S2) and Na loss (see, for instance, analyses 5, 8, 9 and 10, Table S1). Overlap of Ba*L*β on Ce*L*α and Ce*Mζ* on F*K*α were accounted for. The elements Zn, Sr, Gd and U were sought but not detected. Data were reduced using the PAP procedure by Pouchou & Pichoir (1985 ▸).

Analyses show Na loss even under mild conditions (5 nA and 15 µm defocalized beam) with a progressive loss of Na. Also, the formation of cracks on the surface denotes volatilization under vacuum (typical for micro and mesoporous hydrated phases). Totals decrease from 90 wt% to *ca* 86 wt%. Fluorine is absent. Normalization on the basis of 218 charges (see Section 3.4 crystal-chemical formula) using the average of points 1, 2, 3, 4 and 11 (in a separate fragment) gave the empirical formula (Na_9.55_K_1.09_Ba_0.84_Ca_0.08_Th_0.08_)_Σ11.64_(*REE*_1.99_Y_0.01_)_2_(Ti_11.98_Ta_0.05_Nb_0.01_Mn_0.01_Mg_0.01_Fe^2+^_0.01_Zr_0.01_)_Σ12.08_[Si_37.72_Al_0.03_]O_109_, with *REE* = Ce_0.99_La_0.59_Nd_0.27_Pr_0.10_Sm_0.03_Dy_0.01_.

### 2.3. Single-crystal X-ray diffraction   

Single-crystal X-ray diffraction study of ilmajokite was done at the Resource Center ‘X-ray Diffraction Methods’ of St Petersburg State University using the Bruker Kappa APEX DUO diffractometer (microfocus tube) equipped with a CCD area detector. The study was done using monochromatic Mo *K*α X-radiation (λ = 0.71073 Å), with frame widths of 0.5° in ω and 30 s counting time for each frame. The intensity data were reduced and corrected for Lorentz, polarization and background effects using the Bruker software *APEX2* (Bruker-AXS, 2014 ▸). A semiempirical absorption-correction based upon the intensities of equivalent reflections was applied using *SADABS* (Sheldrick, 2007 ▸). The structure was solved and refined in the space group *C*2/*c* to *R*_1_ = 0.081 (*wR*_2_ = 0.233) for 14 797 unique observed reflections with *I* ≥ 2σ(*I*) using the *SHELX* program package (Sheldrick, 2015 ▸) within the *Olex2* shell (Dolomanov *et al.*, 2009 ▸). Crystal data, data collection information and refinement details are given in Table 1 ▸.

The unit-cell parameters of ilmajokite determined in our study correspond well with those reported by Cámara *et al.* (2010 ▸) for their room-temperature study. The most significant difference is for the *c* parameter, which in our study is about 2 Å longer than that determined by Cámara *et al.* (2010 ▸). It is most likely that this difference is due to the different hydration states of the two samples, which also explains the lower quality of the diffraction data for the crystal with the smaller *c* parameter (see Fig. S1).

## 3. Results   

### 3.1. Atom coordination   

The crystal structure of ilmajokite contains 236 symmetrically independent sites, including 1 Ba, 2 *REE* (rare-earth elements, with Ce as the dominant component), 12 Ti, 41 Si, 15 Na, 3 K, 84 O, 38 OH and 40 H_2_O sites. Cation coordination numbers, average bond lengths and their variations, and bond-valence sums are given in Table 2 ▸. The coordination of Ti^4+^ and Si^4+^ cations is octahedral and tetrahedral, as is typical for many other natural and synthetic titanosilicates (Krivovichev, 2005 ▸). The low coordination numbers and bond-valence sums for several alkali-metal (Na, K) sites are due to their low occupancies (up to 0.22). Of 84 O sites, 38 are bridging between adjacent Si atoms; 44 are bridging between Si and Ti atoms; 5 are bonded to Si, Ti and Ce atoms; 6 are bonded to two Ti and one Ce atoms each; one is bonded to two Ti and one Si atoms. Of 38 OH groups, 4 are bonded to two Ti atoms, and the remaining 34 are terminal (silanol) groups of SiO_4_ tetrahedra. The bond-valence sums for the O atoms, OH and H_2_O groups (without contributions from the H atoms) are in the ranges 1.73–2.28, 0.89–1.42 and 0.0–0.58, respectively. The most significant deviations from the expected values (2.00, 1.20 and 0–0.40, respectively) are observed for disordered cation sites that cannot be estimated correctly.

### 3.2. Local topological features   

The crystal structure of ilmajokite is based on a titanosilicate framework of unprecedented complexity. Analysis of the local topological features of the SiO_4_ tetrahedra shows that they belong to ten different types, from *Q* ^2^ (two-connected) to *Q* ^5^ groups [herein, *Q* ^*n*^ indicates a tetrahedron that shares its O corners with *n* adjacent coordination polyhedra (only Si and Ti polyhedra are taken into account)]. The *Q* ^5^ type is not typical for silicate frameworks of corner-sharing tetrahedra (Liebau, 1985 ▸), but occurs in octahedral–tetrahedral frameworks. In ilmajokite, it corresponds to the Si10O_4_ tetrahedron that shares three of its corners with three adjacent tetrahedra and one corner with two TiO_6_ octahedra sharing a common edge (see below). In order to distinguish between silicate tetrahedra of the same type but with different chemical environments, we use the notation *Q* ^*n*^_*m*Ti + *k*Si_, where *m* + *k* = *n*. For instance, there are three types of *Q* ^3^ tetrahedra in ilmajokite, *Q* ^3^_Ti + 2Si_, *Q* ^3^_3Si_ and *Q* ^3^_2Ti + Si_. The complete local topological classification of silicate tetrahedra in ilmajokite is given in Table 3 ▸. It is noteworthy that all *Q* ^2^_2Si_ tetrahedra are only partly occupied with site occupancies less than 0.5.

### 3.3. Structural organization   

**Figure 1:**
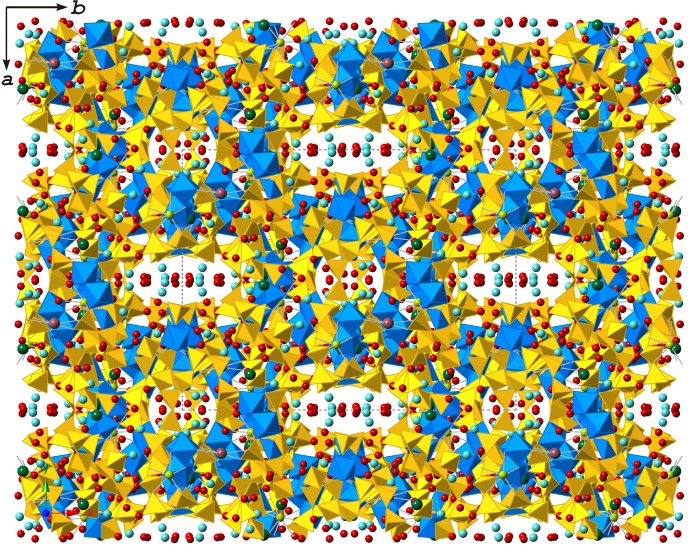
Projection of the crystal structure of ilmajokite along the *c* axis. Legend: Si tetrahedra – yellow, Ti octahedra – blue; H_2_O molecules, Na, K, Ba and Ce atoms are shown as red, light-blue, green, brown and orange spheres, respectively.

**Figure 2:**
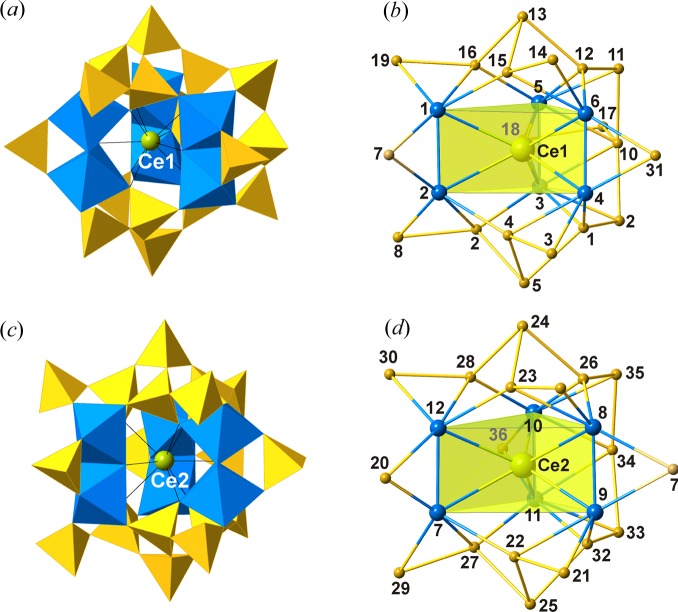
TPTS clusters in the crystal structure of ilmajokite shown in (*a*) and (*c*) polyhedral and (*b*) and (*d*) nodal representations. The numbering scheme corresponds to the numbering of Si and Ti atoms from the experiment. The Ce-centered Ti_6_ trigonal prism is highlighted in yellow. The legend follows that of Fig. 1 ▸.

The projection of the crystal structure of ilmajokite along the *c* axis is shown in Fig. 1 ▸. The titanosilicate framework has a complex organization that can be described as follows. Two TiO_6_ octahedra share a common edge to form a [Ti_2_O_10_] dimer. Three dimers with parallel orientation form a trigonal prism centered by Ce^3+^ in [9]-coordination. The triple-dimer titanate structure is surrounded by SiO_4_ tetrahedra to form a trigonal prismatic titanosilicate cluster, further denoted as a TPTS cluster. There are two symmetrically different TPTS clusters centered by the Ce1 and Ce2 atoms [Figs. 2 ▸(*a*) and 2(*c*)]. For further description of the structural topology of the framework, we adopt a nodal representation, where each Ti and Si polyhedron is symbolized by a node of respective color and two nodes are linked by an edge if the two corresponding polyhedra share a common O atom. This approach is widely used for the description of complex topologies observed in zeolite-type tetrahedral (Baerlocher *et al.*, 2007 ▸; Smith, 2000 ▸) and heteropolyhedral (Krivovichev, 2005 ▸; Krivovichev *et al.*, 2005 ▸) frameworks. The nodal representation of the two independent TPTS clusters in ilmajokite is given in Figs. 2 ▸(*b*) and 2(*d*).

**Figure 3:**
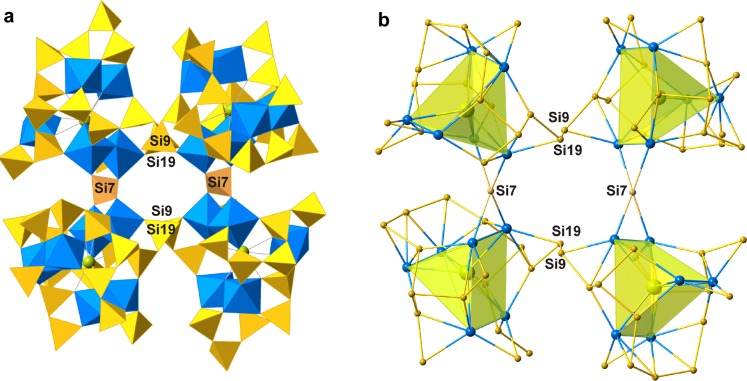
Four-membered ring of the TPTS clusters shown in (*a*) polyhedral and (*b*) nodal representations. Legend and numbering scheme follow that of Fig. 1 ▸.

**Figure 4:**
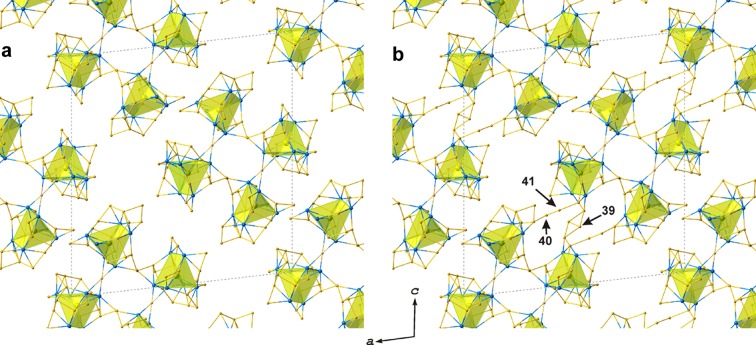
(*a*) Arrangement of the four-membered rings of the TPTS clusters within the (010) plane and (*b*) their linkage through additional Si39, Si40 and Si41 nodes into ribbons.

**Figure 5:**
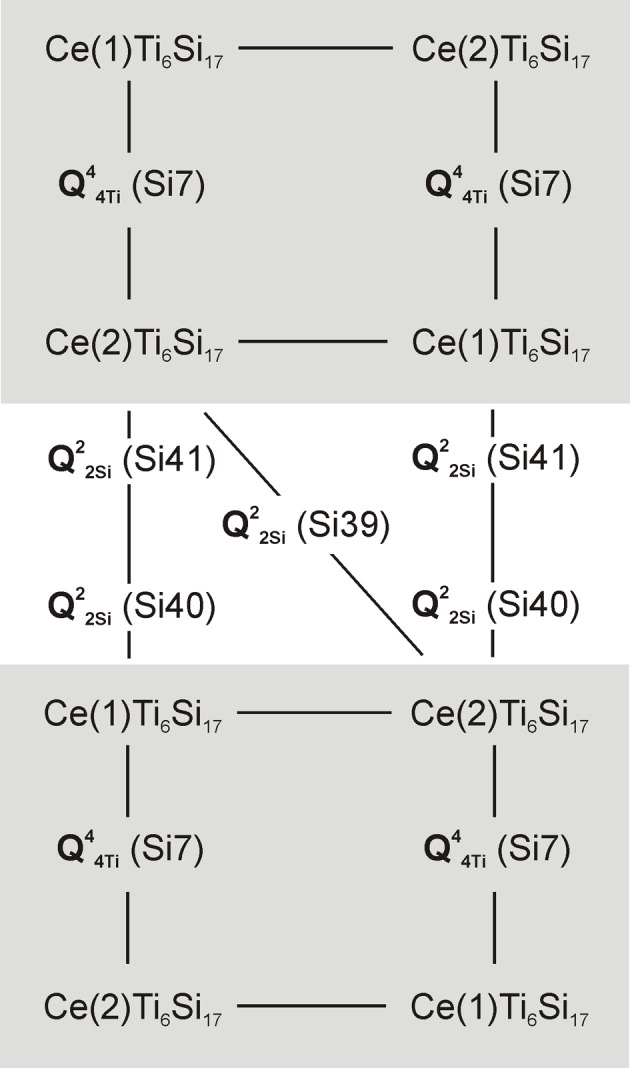
Schematic representation of the topology of ribbons formed by TPTS (CeTi_6_Si_17_) clusters in ilmajokite (four-membered rings are highlighted in gray).

**Figure 6:**
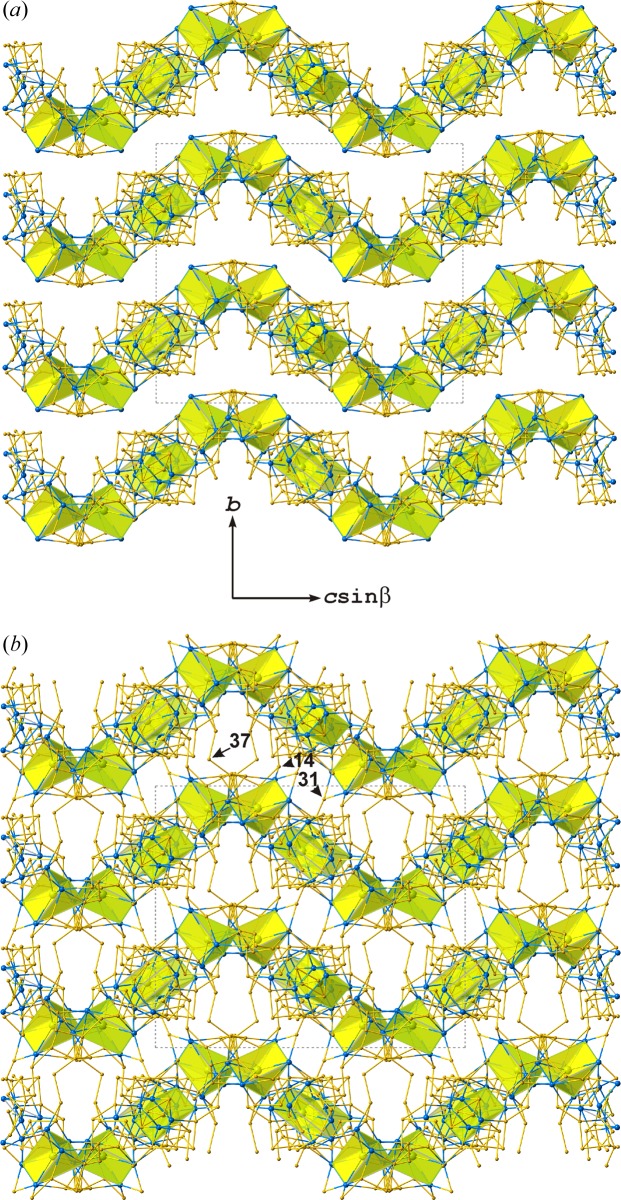
(*a*) Arrangement of layers of ribbons of TPTS clusters along the *b* axis and (*b*) their linkage into a 3D framework via Si14, Si31 and Si37 nodes.

Four adjacent TPTS clusters are linked *via* the Si9–Si19 links and the Si7O_4_ tetrahedron (note that this is the only Q^4^_4Ti_ tetrahedron in the crystal structure) to form a four-membered ring (Fig. 3 ▸). The rings are arranged within the (010) plane [Fig. 4 ▸(*a*)] and linked *via* partly occupied Si39, Si40 and Si41 polyhedra (*Q* ^2^_2Si_ type) into ribbons parallel to 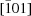 [Fig. 4 ▸(*b*)]. A schematic description of the topology of the chain is given in Fig. 5 ▸. The ribbons are organized into layers parallel to (010). The view of the layers along the *a* axis [Fig. 6 ▸(*a*)] shows that they are modulated with a modulation wavelength of *c*sinβ = 32.91 Å and an amplitude of ∼*b*/2 = 13.89 Å. The layers are linked *via* Si14, Si14 and Si31 tetrahedra; with the first two partly occupied (the *Q* ^2^_2Si_ type) and the last fully occupied (the *Q* ^3^_2Ti + Si_ type). Thus the most condensed unit in the titanosilicate framework is the four-membered ring of the TPTS clusters, whereas the linkage of the rings proceeds *via* Si14, Si31, Si37, Si38, Si39, Si40 and Si41 tetrahedra; with only Si31 fully occupied, whereas the others are less than half-occupied.

Na^+^, K^+^, Ba^2+^ and H_2_O groups occur in the framework cavities and have different occupancies and coordination environments (Table 2 ▸).

It is noteworthy that the mosaicity observed in the ILM02 crystal has lower resolution in the direction of the ribbons, reflecting damage in the structure of that crystal, probably related to amorphization by dehydration.

### 3.4. Crystal-chemical formula   

The crystal-chemical formula determined from the structure refinement is Na_11.24_K_1.10_Ba_0.90_Ce_2_Ti_12_Si_37.52_O_94_(OH)_30.38_·(H_2_O)_29.06_, in close agreement between the sums of the positive (+218.22) and negative (−218.38) charges, which is remarkable in view of the difficulties associated with the structure refinement. There is also a very good agreement with the results of the electron-microprobe analysis (see Section 2.2), particularly considering the difficulty due to dehydration and Na migration under the beam. Assuming that the selected points with less Na are closer to the actual H_2_O content, the formula is in accordance with 14 H_2_O groups per formula unit and the following empirical formula: (Na_9.55_K_1.09_Ba_0.84_Ca_0.08_Th_0.08_)_Σ11.64_(*REE*_1.99_Y_0.01_)_2_(Ti_11.96_Ta_0.05_Nb_0.01_Mn_0.01_Mg_0.01_Fe^2+^_0.01_Zr_0.01_)_Σ12.06_[Si_37.68_Al_0.03_]O_94_(OH)_30_·(H_2_O)_14.18_, with *REE* = Ce_0.99_La_0.59_Nd_0.27_Pr_0.10_Sm_0.03_Dy_0.01._

Taking into account the presence of the titanosilicate framework and its silicate sub-framework, the detailed crystal-chemical formula of ilmajokite may be written as Na_11.24_K_1.10_Ba_0.90_〈Ce_2_{Ti_12_[Si_37.52_O_88_(OH)_26.38_]O_6_(OH)_4_}〉·(H_2_O)_29.06_, where square, curly and angular brackets denote silicate, titanosilicate and rare-earth-titanosilicate substructures. On the basis of both chemical and structural data, the ideal crystal-chemical formula of ilmajokite can be written as Na_11_KBaCe_2_Ti_12_Si_37.5_O_94_(OH)_31_^.^29H_2_O, which requires SiO_2_ 46.06, TiO_2_ 19.60, Ce_2_O_3_ 6.69, BaO 3.14, Na_2_O 6.97, K_2_O 0.96, H_2_O 16.58 (total 100 wt%). The amount of H_2_O in the crystal-chemical formula disagrees with the value of 24.54 wt% reported by Bussen *et al.* (1972 ▸), which could be due to the possibility of variable hydration states frequently observed for microporous framework minerals. The two-step dehydration described by Bussen *et al.* (1972 ▸) may correspond to the loss of zeolitic H_2_O and the complete dehydration associated with the hydroxyl groups.

The ideal formula of the silicate sub-framework, assuming full occupancy of the partly occupied Si and associated OH sites, and excluding the Si37–Si38 disorder, can be written as [Si_40_O_88_(OH)_32_]^48−^ or [Si_5_O_11_(OH)_4_]^6−^ with the amazingly simple Si:O ratio of 1:3. It is interesting to note that the Si:O 1:3 ratio has previously been reported for other rather complex silicate minerals such as hyttsjöite, Pb_18_Ba_2_Ca_5_Mn_2_^2+^Fe_2_^3+^[Si_30_O_90_]Cl(H_2_O)_6_ (Grew *et al.*, 1996 ▸); aerinite, Ca_6_FeAl(Fe,Mg)_2_(Al,Mg)_6_[Si_12_O_36_](OH)_12_H)(H_2_O)_12_(CO_3_) (Rius *et al.*, 2004 ▸, 2009 ▸); and sveinbergeite, (H_2_O)_2_(Ca(H_2_O))(Fe_6_^2+^Fe^3+^)Ti_2_[Si_4_O_12_]_2_O_2_(OH)_4_((OH)H_2_O)) (Khomyakov *et al.*, 2011 ▸). We note that the silicate subframework found in the crystal structure of ilmajokite is new and has not been observed previously in any mineral or inorganic compound (Pushcharovsky *et al.*, 2016 ▸).

## 4. Discussion   

### 4.1. Hierarchical analysis   

**Figure 7:**
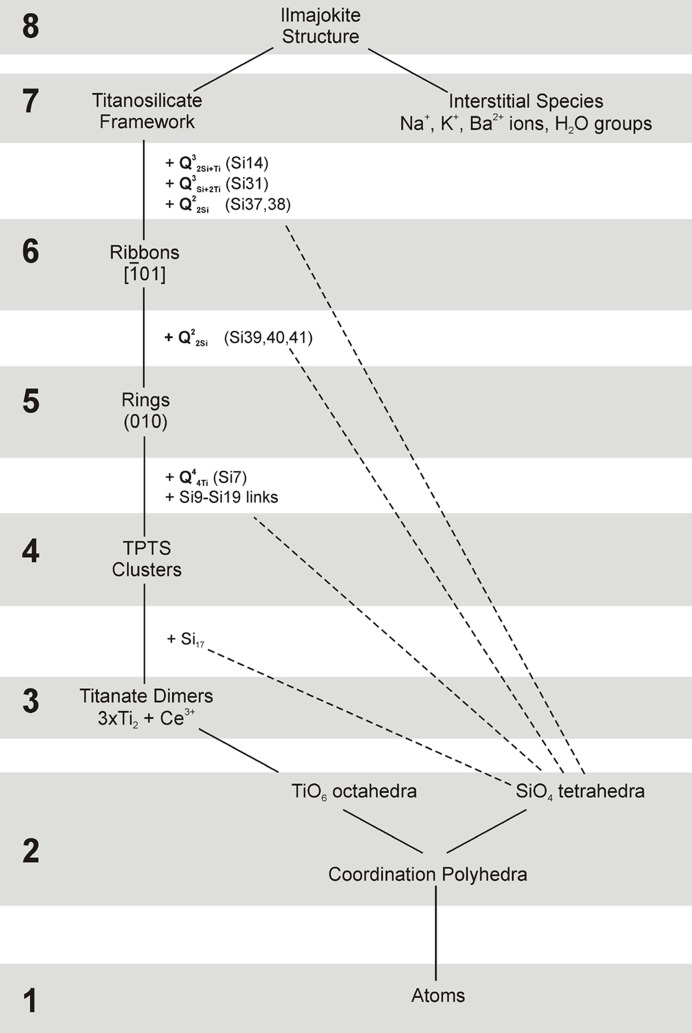
Hierarchial organization of the crystal structure of ilmajokite separated into eight hierarchy levels (highlighted in gray).

The economist and Nobel laureate Herbert Simon once noted that ‘hierarchy… is one of the central structural schemes that the architect of complexity uses’ (Simon, 1962 ▸). The high number of hierarchical levels of structural organization (hierarchical depth) reflects the high degree of complexity of a system. As has been noted previously [see, *e.g.* Makovicky (1997 ▸); Ferraris *et al.* (2004 ▸); Hawthorne (2014 ▸)], the crystal structures of minerals and inorganic compounds have a hierarchical organization [see Krivovichev (2017 ▸) for a detailed discussion and examples]. From this point of view, the crystal structure of ilmajokite possesses a multilevel hierarchical structure (Fig. 7 ▸). The first (lowest) level consists of atoms that are grouped into coordination polyhedra (second level). The TiO_6_ octahedra are linked to form dimers (third level). The dimers together with SiO_4_ tetrahedra and centering Ce^3+^ cations comprise TPTS clusters (fourth level). The clusters are linked to form four-membered rings (fifth level), which are further interlinked to form ribbons (sixth level). The ribbons are united into a three-dimensional octahedral–tetrahedral framework (seventh level), which, together with alkali metal, Ba^2+^ cations and H_2_O molecules complete the structure organization (eighth level). The subdivision of the structure into eight levels reflects its high complexity which is considered in the next section.

### 4.2. Complexity analysis   

The complexity of the crystal structure of ilmajokite can be quantitatively estimated using information-based complexity measures recently proposed by Krivovichev (2012 ▸, 2013 ▸, 2014 ▸). Since the positions of the H atoms were not determined from the single-crystal diffraction experiment, the procedure of H-correction was applied as described by Pankova *et al.* (2018 ▸). The resulting parameters are as follows: the number of atoms per reduced unit cell, *v*, is equal to 1416; the amount of Shannon information per atom, *I*_G_, is 8.468 bits; and the amount of Shannon information per unit cell, *I*_G, total_, is 11 990.129 bits. The parameters without H-corrections are *v* = 972, *I*_G_ = 7.925 bits per atom, *I*_G, total_ = 7702.918 bits per cell, which means that hydration is responsible for *ca* 36% of the total structural complexity. The total value of Shannon information places ilmajokite as the third-most complex mineral known to date after ewingite, Mg_4_Ca_4_(UO_2_)_12_(CO_3_)_15_O_2_(OH)_6_·69H_2_O [23 477.507 bits per cell with H-correction and 12 684.86 bits per cell without H-correction (∼46% of total complexity is due to H atoms); Olds *et al.* (2017 ▸)] and morrisonite, Ca_11_(As^3+^V_2_^4+^V_10_^5+^As_6_^5+^O_51_)_2_·78H_2_O [13 588.350 bits per cell with H-correction and 7553.229 bits per cell after H-correction (H atoms are responsible for ∼44% of structural complexity); Kampf *et al.* (2016 ▸)]. Since both ewingite and morrisonite contain nanoscale clusters, ilmajokite is the most complex with a framework structure, and in terms of its complexity, is very close to paulingite-(Ca), Ca_5_(Al_10_Si_32_O_84_)·34H_2_O [11 590.532 bits per cell with H-correction and 6766.998 bits per cell after H-correction (∼42% complexity is due to H atoms); Passaglia *et al.* (2001 ▸)].

## 5. Conclusions   

Under natural conditions, ilmajokite forms as one of the latest minerals of hydrothermal activity, which includes several stages and a range of precursor phases that precede the crystallization of the mineral. The extreme complexity of ilmajokite is the result of a combination of a number of factors, including its high chemical complexity and seggregation of chemically different elements (Na, K, Ba, Ce, Ti, Si) into their own crystallographic sites (the most interesting is the absence of any detectable K–Ba substitution), the presence of polynuclear TPTS clusters of nanoscale size (the diameter of the cluster is around 1.4 nm), condensation of the TPTS clusters into larger four-membered units, the high hydration state, *etc*.

## Supplementary Material

Crystal structure: contains datablock(s) I. DOI: 10.1107/S2052252519016622/lt5023sup1.cif

Structure factors: contains datablock(s) I. DOI: 10.1107/S2052252519016622/lt5023Isup2.hkl

Supplementary data. DOI: 10.1107/S2052252519016622/lt5023sup3.pdf

CCDC reference: 1971431

## Figures and Tables

**Table 1:** Crystal data and structure refinement for ilmajokite

Crystal data	
Chemical formula	Ba_0.45_H_44.25_Ce_1.04_K_0.55_Na_5.62_O_76.74_Si_18.76_Ti_6_
*M*_r_	2445.37
Crystal system, space group	Monoclinic, *C*2/*c*
Temperature (K)	296
*a*, *b*, *c* (Å)	35.908 (5), 27.784 (3), 33.126 (4)
β (°)	96.494 (3)
*V* (Å^3^)	32836 (7)
*Z*	16
Radiation type	Mo *K*α
μ (mm^−1^)	1.79
Crystal size (mm)	0.15 × 0.08 × 0.04

Data collection	
Diffractometer	Bruker Kappa APEX DUO
Absorption correction	Multi-scan
No. of measured, independent, observed [*I* > 2σ(*I*)] reflections	108441, 21527, 14797
*R*_int_	0.130
θ_max_ (°)	22.7
(sin θ/λ)_max_ (Å^−1^)	0.543

Refinement	
*R*[*F* ^2^ > 2σ(*F* ^2^)], *wR*(*F* ^2^), *S*	0.081, 0.264, 0.97
No. of parameters	1847
No. of restraints	18
H-atom treatment	H-atom parameters not defined
	*w* = 1/[σ^2^(*F*_o_^2^) + (0.1738*P*)^2^ + 608.3564*P*] where *P* = (*F*_o_^2^ + 2*F*_c_^2^)/3
Δρ_max_, Δρ_min_ (e Å^−3^)	1.89, −2.80

**Table 2:** Coordination numbers (CNs) of cations, average bond lengths and their variations (Å), and bond-valence sums (BVS, in valence units, v.u.) for the crystal structure of ilmajokite Bond valence units were calculated using parameters from Gagné & Hawthorne (2015 ▸).

Atom	CN	Bond lengths		BVS
		Average	Range (min–max)	
Ba	10	2.899	2.818–2.968	1.91
K	6, 7	2.816	2.403–3.308	1.03–1.27
Na	3, 5, 6, 7, 8	2.555	1.999–3.022	0.42–1.10
Ce	9	2.543	2.481–2.626	3.00
Ti	6	1.955	1.853–2.105	3.99–4.17
Si	4	1.621	1.512–1.671	3.87–4.27

**Table 3:** Topological types of Si tetrahedra in the crystal structure of ilmajokite

General type	Specific type	Si Sites
*Q* ^2^	*Q* ^2^_Si + Ti_	3, 21
	*Q* ^2^_2Si_	17†, 37†, 38†, 39†, 40†, 41†
	*Q* ^2^_2Ti_	36
*Q* ^3^	*Q* ^3^_2Si + Ti_	2, 8, 9, 14, 19, 29, 33, 35
	*Q* ^3^_3Si_	5, 13, 24, 25
	*Q* ^3^_2Ti + Si_	20, 31
*Q* ^4^	*Q* ^4^_2Ti + 2Si_	1, 4, 6, 12, 15, 16, 18, 22, 23, 26, 27, 28, 32, 34
	*Q* ^4^_4Ti_	7
	*Q* ^4^_3Si + Ti_	11, 30
*Q* ^5^	*Q* ^5^_3Si + 2Ti_	10

† Partially occupied sites.

## References

[bb1] Andrade, M. B., Yang, H., Downs, R. T., Färber, G., Contreira Filho, R. R., Evans, S. H., Loehn, C. W. & Schumer, B. N. (2018). *Miner. Mag.* **82**, 121–131.

[bb2] Anthony, R. G., Dosch, R. G., Gu, D. & Philip, C. V. (1994). *Ind. Eng. Chem. Res.* **33**, 2702–2705.

[bb3] Baerlocher, Ch., McCusker, L. B. & Olson, D. H. (2007). *Atlas of Zeolite Framework Types*, 6th ed. Amsterdam: Elsevier.

[bb4] Bruker (2014). *APEX2.* Bruker AXS Inc., Madison, Wisconsin, USA.

[bb5] Bussen, I. V., Gannibal, L. F., Goiko, E. A., Mer’kov, A. N. & Nedorezova, A. P. (1972). *Zapiski Vserossi -Bciskogo mineralogicheskogo obshchestva (Proceedings of the Russian Mineralogical Society)*, **101**, 75–79 .

[bb6] Cámara, F., Bindi, L., Tribaudino, M., Vescovi, F. & Bacchi, A. (2010). *89th SIMP Meeting Programme and Book of Abstracts*, Ferrara, 13–15 September 2010, p. 258. Società Italiana di Mineralogia e Petrografia.

[bb7] Cámara, F., Sokolova, E., Abdu, Y. A., Hawthorne, F. C., Charrier, T., Dorcet, V. & Carpentier, J.-F. (2017). *Miner. Mag.* **81**, 369–381.

[bb8] Cuko, A., Calatayud, M. & Bromley, S. T. (2018). *Nanoscale* **10**, 832–842. 10.1039/c7nr05758j 29261197

[bb9] Dolomanov, O. V., Bourhis, L. J., Gildea, R. J., Howard, J. A. K. & Puschmann, H. (2009). *J. Appl. Cryst.* **42**, 339–341.

[bb10] Ferraris, G., Makovicky, E. & Merlino, S. (2004). *Crystallography of Modular Materials.* Oxford University Press.

[bb11] Figueiredo, B. R., Cardoso, S. P., Portugal, I., Rocha, J. & Silva, C. M. (2018). *Sep. Purif. Rev.* **47**, 306–336.

[bb12] Gagné, O. C. & Hawthorne, F. C. (2015). *Acta Cryst.* B**71**, 562–578. 10.1107/S2052520615016297 PMC4591556 26428406

[bb13] Goiko, E. A., Bussen, I. V., Gannibal, L. F. & Lipatova, E. A. (1974). *Uch. Zap. Leningr. Gos. Univ. Ser. Geol. Nauk*, **278**, 174–181.

[bb14] Grew, E. S., Peacor, D. R., Rouse, R. C., Yates, M. C., Su, S.-C. & Marquez, N. (1996). *Am. Mineral.* **81**, 743–753.

[bb15] Harrison, W. T. A., Gier, T. E. & Stucky, G. D. (1995). *Zeolites*, **15**, 408–412.

[bb16] Hawthorne, F. C. (2014). *Miner. Mag.* **78**, 957–1027.

[bb17] Kampf, A. R., Hughes, J. M., Nash, B. P. & Marty, J. (2016). *Can. Mineral.* **54**, 145–162.

[bb18] Khomyakov, A. P. (1994). *Zapiski Vserossi -Bciskogo mineralogicheskogo obshchestva (Proceedings of the Russian Mineralogical Society)*, **123**, 40–43.

[bb19] Khomyakov, A. P., Cámara, F., Sokolova, E., Abdu, Y. & Hawthorne, F. C. (2011). *Miner. Mag.* **75**, 2687–2702.

[bb20] Krivovichev, S. V. (2005). *Rev. Mineral. Geochem.* **57**, 17–68.

[bb21] Krivovichev, S. (2012). *Acta Cryst.* A**68**, 393–398. 10.1107/S0108767312012044 22514071

[bb22] Krivovichev, S. V. (2013). *Miner. Mag.* **77**, 275–326.

[bb23] Krivovichev, S. V. (2014). *Angew. Chem. Int. Ed.* **53**, 654–661. 10.1002/anie.201304374 24339343

[bb24] Krivovichev, S. V. (2017). *Crystallogr. Rev.* **23**, 2–71.

[bb25] Krivovichev, S. V., Cahill, C. L., Nazarchuk, E. V., Burns, P. C., Armbruster, T. & Depmeier, W. (2005). *Microporous Mesoporous Mater.* **78**, 209–215.

[bb26] Krivovichev, S. V., Yakovenchuk, V. N., Armbruster, T., Döbelin, N., Pattison, P., Weber, H.-P. & Depmeier, W. (2004). *Am. Mineral.* **89**, 1561–1565.

[bb27] Kuznicki, S. M., Bell, V. A., Nair, S., Hillhouse, H. W., Jacubinas, R. M., Braunbarth, C. M., Toby, B. H. & Tsapatsis, M. (2001). *Nature*, **412**, 720–724. 10.1038/35089052 11507636

[bb28] Liebau, F. (1985). *Structural Chemistry of Silicates: Structure, Bonding and Classification.* Berlin: Springer-Verlag.

[bb29] Lykova, I. S., Chukanov, N. V., Pekov, I. V., Yapaskurt, V. O. & Giester, G. (2018). *Eur. J. Mineral.* **30**, 289–304.

[bb30] Makovicky, E. (1997). *Modular Aspects of Minerals. European Mineralogical Union Notes in Mineralogy*, Vol 1, edited by S. Merlino, pp. 315–343. Budapest: Eötvös University Press.

[bb31] Men’shikov, Y. P., Sokolova, E. V., Egorov-Tismenko, Y. K., Khomyakov, A. P. & Polezhaeva, L. I. (1992). *Zapiski Vserossi -Bciskogo mineralogicheskogo obshchestva (Proceedings of the Russian Mineralogical Society)*, **121**, 94–99.

[bb32] Mer’kov, A. N., Bussen, I. V., Goiko, E. A., Kul’chitskaya, E. A., Men’shikov, Yu. P. & Nedorezova, A. P. (1973). *Zapiski Vserossi -Bciskogo mineralogicheskogo obshchestva (Proceedings of the Russian Mineralogical Society)*, **102**, 54–62.

[bb33] Milyutin, V. V., Nekrasova, N. A., Yanicheva, N. Y., Kalashnikova, G. O. & Ganicheva, Y. Y. (2017). *Radiochemistry*, **59**, 65–69.

[bb34] Noh, Y. D., Komarneni, S. & Mackenzie, K. J. D. (2012). *Sep. Purif. Technol.* **95**, 222–226.

[bb35] Olds, T. A., Plášil, J., Kampf, A. R., Simonetti, A., Sadergaski, L. R., Chen, Y. S. & Burns, P. C. (2017). *Geology*, **45**, 1007–1010.

[bb36] Oleksiienko, O., Wolkersdorfer, C. & Sillanpää, M. (2017). *Chem. Eng. J.* **317**, 570–585.

[bb37] Pakhomovsky, Y. A., Panikorovskii, T. L., Yakovenchuk, V. N., Ivanyuk, G. Y., Mikhailova, J. A., Krivovichev, S. V., Bocharov, V. N. & Kalashnikov, A. O. (2018). *Eur. J. Mineral.* **30**, 525–535.

[bb38] Pankova, Y. A., Gorelova, L. A., Krivovichev, S. V. & Pekov, I. V. (2018). *Eur. J. Mineral.* **30**, 277–287.

[bb39] Passaglia, E., Gualtieri, A. & Marchi, E. (2001). *Eur. J. Mineral.* **13**, 113–119.

[bb40] Pekov, I. V., Zubkova, N. V., Yapaskurt, V. O., Belakovskiy, D. I., Lykova, I. S., Britvin, S. N., Turchova, A. G. & Pushcharovsky, D. Y. (2019). *Eur. J. Mineral.* **31**, 557–564.

[bb41] Pekov, I. V. (2001). *Lovozero Massif: History, Pegmatites, Minerals.* Moscow: Ocean Pictures Ltd.

[bb42] Pouchou, J. L. & Pichoir, F. (1985). In *Microbeam analysis*, edited by J. T. Armstrong, pp. 104–106. San Francisco Press.

[bb43] Přech, J. (2018). *Catal. Rev.* **60**, 71–131.

[bb61] Pushcharovsky, D. Y., Zubkova, N. V. & Pekov, I. V. (2016). *Struct. Chem.* **26**, 1593–1603.

[bb44] Rius, J., Crespi, A., Roig, A., Melgarejo, J. C. & Carles, (2009). *Eur. J. Mineral.* **21**, 233–240.

[bb45] Rius, J., Elkaim, E. & Torrelles, X. (2004). *Eur. J. Mineral.* **16**, 127–134.

[bb46] Rocha, J. & Anderson, M. W. (2000). *Eur. J. Inorg. Chem.* **2000**, 801–818.

[bb47] Rozhdestvenskaya, I. V., Kogure, T., Abe, E. & Drits, V. A. (2009). *Miner. Mag.* **73**, 883–890.

[bb48] Rozhdestvenskaya, I. V., Mugnaioli, E., Czank, M., Depmeier, W., Kolb, U., Reinholdt, A. & Weirich, T. (2010). *Miner. Mag.* **74**, 159–177.

[bb49] Rozhdestvenskaya, I. V., Mugnaioli, E., Schowalter, M., Schmidt, M. U., Czank, M., Depmeier, W. & Rosenauer, A. (2017). *IUCrJ*, **4**, 223–242. 10.1107/S2052252517002585 PMC5414397 28512570

[bb50] Selivanova, E. A., Lyalina, L. M. & Savchenko, Y. E. (2018). *Minerals*, **8**, 458.

[bb51] Sheldrick, G. M. (2007). *SADABS.* University of Goettingen, Germany.

[bb52] Sheldrick, G. M. (2015). *Acta Cryst.* C**71**, 3–8.

[bb53] Simon, H. A. (1962). *Proc. Am. Philos. Soc.* **106**, 467–482.

[bb54] Smith, J. V. (2000). *Microporous and other Framework Materials with Zeolite-Type Structures. Subvol. A. Tetrahedral Frameworks of Zeolites, Clathrates and Related Materials*, Vol 14A, edited by W. H. Baur & R. X. Fischer. Berlin Heidelberg: Springer-Verlag.

[bb55] Sokolova, E. & Cámara, F. (2017). *Miner. Mag.* **81**, 1457–1484.

[bb56] Sokolova, E., Cámara, F., Hawthorne, F. C., Semenov, E. I. & Ciriotti, M. E. (2017). *Miner. Mag.* **81**, 175–181.

[bb57] Sokolova, E. V., Rastsvetaeva, R. K., Andrianov, V. I., Egorov-Tismenko, Y. K. & Men’shikov, Y. P. (1989). *Sov. Phys. Dokl.* **34**, 583–585.

[bb58] Yakovenchuk, V. N., Nikolaev, A. P., Selivanova, E. A., Pakhomovsky, Y. A., Korchak, J. A., Spiridonova, D. V., Zalkind, O. A. & Krivovichev, S. V. (2009). *Am. Mineral.* **94**, 1450–1458.

[bb59] Zhitova, E. S., Zolotarev, A. A., Hawthorne, F. C., Krivovichev, S. V., Yakovenchuk, V. N. & Goncharov, A. G. (2019). *Acta Cryst.* B**75**, 578–590. 10.1107/S2052520619006024 32830715

[bb60] Zolotarev, A. A., Selivanova, E. A., Krivovichev, S. V., Savchenko, Y. E., Panikorovskii, T. L., Lyalina, L. M., Pautov, L. A. & Yakovenchuk, V. N. (2018). *Minerals*, **8**, 303.

