# Supplementary material for: Extraordinary structural complexity of ilmajokite: a multilevel hierarchical framework structure of natural origin
Source: IUCrJ. 2020 Jan 1;7(Pt 1):121–8. doi: 10.1107/S2052252519016622 (PMC6949600; doi:10.1107/S2052252519016622)
Supplement: Supplementary file 3 [file m-07-00121-sup3.pdf]

IUCrJ

Volume 7 (2020)

Supporting information for article:

Extraordinary structural complexity of ilmajokite: a multilevel hierarchical framework structure of natural origin

Andrey A. Zolotarev, Sergey V. Krivovichev, Fernando Cámara, Luca Bindi, Elena S. Zhitova, Frank Hawthorne and Elena Sokolova

Table S1. EMPA analyses if ilmajokite (normalized on the basis of 109 O pfu)

point	1	2	3	4	5	6	7	8	9	10	11	average	average*	average**
SiO_2	48.45	50.00	50.73	49.01	51.32	52.05	49.72	52.38	52.00	52.14	49.47	50.66	49.53	49.53
Al_2O_3	0.02	0.04	0.03	0.06	0.05	0.04	0.02	0.01	0.02	0.04	0.00	0.03	0.03	0.03
TiO_2	21.07	20.84	22.05	20.60	21.27	21.69	21.68	22.49	22.67	21.80	20.03	21.47	20.92	20.92
FeO	0.01	0.01	0.02	0.00	0.00	0.00	0.00	0.07	0.03	0.03	0.00	0.02	0.01	0.01
MnO	0.02	0.00	0.02	0.00	0.04	0.02	0.00	0.00	0.00	0.03	0.01	0.01	0.01	0.01
MgO	0.01	0.01	0.01	0.01	0.01	0.01	0.01	0.01	0.01	0.01	0.01	0.01	0.01	0.01
CaO	0.10	0.08	0.10	0.09	0.08	0.06	0.07	0.10	0.10	0.10	0.12	0.09	0.10	0.10
BaO	2.98	2.89	2.75	2.52	3.22	3.18	3.15	3.41	3.30	3.00	2.89	3.03	2.80	2.80
$Na_2O^{\sim}$	7.29	5.18	4.93	6.71	2.66	3.27	3.33	1.37	1.37	0.68	8.22	4.09	6.47	6.47
K_2O	1.00	1.26	1.12	1.03	1.21	1.07	1.09	0.97	0.89	0.78	1.20	1.06	1.12	1.12
Nb_2O_5	0.00	0.07	0.08	0.00	0.05	0.07	0.11	0.00	0.00	0.42	0.00	0.08	0.03	0.03
Ta_2O_5	0.00	0.19	0.37	0.20	0.00	0.05	0.07	0.05	0.17	0.19	0.00	0.13	0.15	0.15
La_2O_3	1.89	2.29	2.02	2.17	1.76	2.55	1.83	2.34	2.08	2.41	2.08	2.13	2.09	2.09
Ce_2O_3	3.36	3.36	3.95	3.42	3.87	3.64	3.23	3.74	3.58	3.28	3.69	3.56	3.56	3.56
Pr2O3	0.05	0.55	0.39	0.52	0.54	0.00	0.20	0.33	0.36	0.41	0.22	0.32	0.35	0.35
Nd_2O_3	0.86	1.11	1.16	0.90	0.69	0.87	0.62	0.74	1.15	0.63	0.88	0.87	0.98	0.98
Sm_2O_3	0.03	0.19	0.30	0.03	0.16	0.00	0.00	0.25	0.00	0.19	0.01	0.10	0.11	0.11
Dy_2O_3	0.28	0.00	0.00	0.00	0.00	0.51	0.01	0.03	0.11	0.00	0.00	0.09	0.06	0.06
ZrO_2	0.00	0.00	0.00	0.04	0.03	0.03	0.03	0.00	0.02	0.00	0.06	0.02	0.02	0.02
ThO_2	0.56	0.41	0.49	0.48	0.48	0.39	0.46	0.43	0.27	0.54	0.34	0.44	0.46	0.46
Y_2O_3	0.02	0.02	0.02	0.00	0.06	0.00	0.07	0.00	0.07	0.00	0.00	0.02	0.01	0.01
F	0.00	0.01	0.02	0.00	0.00	0.00	0.00	0.07	0.03	0.03	0.00	0.02	0.01	0.01
H_2O*														11.50
TOTAL	87.98	88.50	90.56	87.78	87.51	89.50	85.71	88.80	88.22	86.72	89.25	88.25	88.82	100.32
O=F	0.00	0.01	0.01	0.00	0.00	0.00	0.00	0.03	0.01	0.01	0.00	0.01	0.00	0.00
TOTAL	87.98	88.50	90.55	87.78	87.51	89.50	85.71	88.77	88.21	86.70	89.25	88.25	88.81	100.31
Si	37.338	38.093	37.765	37.703	38.938	38.742	38.418	38.975	38.861	39.357	37.686	38.350	37.718	37.718
Al	0.018	0.035	0.030	0.059	0.042	0.035	0.014	0.007	0.016	0.038	0.000	0.027	0.028	0.028
Σ T	37.356	38.128	37.795	37.762	38.980	38.777	38.432	38.981	38.877	39.395	37.686	38.377	37.746	37.746
Ti	12.212	11.941	12.346	11.917	12.136	12.140	12.601	12.585	12.742	12.377	11.475	12.224	11.979	11.979
Nb	0.000	0.023	0.026	0.000	0.018	0.023	0.039	0.000	0.000	0.142	0.002	0.027	0.010	0.010
Sn^{4+}	0.000	0.000	0.000	0.000	0.000	0.000	0.000	0.000	0.000	0.000	0.000	0.000	0.000	0.000
Ta	0.000	0.058	0.113	0.062	0.000	0.016	0.021	0.014	0.050	0.059	0.000	0.039	0.047	0.047
Fe^{3+}	0.000	0.000	0.000	0.000	0.000	0.000	0.000	0.000	0.000	0.000	0.000	0.000	0.000	0.000
Fe^{2+}	0.003	0.008	0.015	0.000	0.000	0.000	0.000	0.044	0.018	0.022	0.000	0.010	0.005	0.005
Mn^{2+}	0.015	0.000	0.013	0.000	0.026	0.011	0.000	0.000	0.000	0.017	0.009	0.008	0.007	0.007
Mg	0.007	0.007	0.007	0.007	0.007	0.007	0.007	0.007	0.007	0.007	0.007	0.007	0.007	0.007
Zr	0.000	0.000	0.000	0.015	0.012	0.012	0.013	0.000	0.008	0.000	0.024	0.008	0.008	0.008
Na	10.900	7.658	7.114	10.007	3.916	4.719	4.996	1.970	1.986	0.995	12.141	6.007	9.549	9.549
La	0.537	0.642	0.555	0.616	0.492	0.701	0.522	0.642	0.573	0.671	0.583	0.594	0.587	0.587
Ce^{3+}	0.948	0.937	1.076	0.964	1.074	0.991	0.915	1.019	0.979	0.907	1.030	0.986	0.992	0.992
Pr	0.013	0.153	0.105	0.146	0.148	0.000	0.055	0.089	0.098	0.112	0.062	0.089	0.096	0.096
Nd	0.238	0.301	0.309	0.248	0.187	0.231	0.171	0.198	0.306	0.170	0.240	0.236	0.268	0.268
Sm	0.007	0.049	0.077	0.007	0.043	0.000	0.000	0.065	0.000	0.048	0.002	0.027	0.029	0.029

Dy	0.069	0.000	0.000	0.000	0.000	0.123	0.003	0.008	0.026	0.000	0.000	0.021	0.014	0.014
Ca	0.082	0.066	0.079	0.076	0.063	0.048	0.061	0.080	0.076	0.077	0.097	0.073	0.080	0.080
Th	0.098	0.071	0.083	0.083	0.083	0.066	0.080	0.073	0.045	0.093	0.059	0.076	0.079	0.079
K	0.981	1.220	1.060	1.007	1.176	1.012	1.074	0.923	0.847	0.752	1.169	1.019	1.088	1.088
Ba	0.899	0.864	0.801	0.759	0.958	0.926	0.954	0.996	0.967	0.887	0.862	0.898	0.837	0.837
Y	0.009	0.008	0.006	0.000	0.025	0.000	0.031	0.000	0.029	0.000	0.000	0.010	0.005	0.005
F	0.000	0.031	0.055	0.000	0.000	0.000	0.000	0.166	0.068	0.081	0.000	0.041	0.018	0.018
OH														58.463

* Average of analyses 1,2,3,4,11. ** Average of analyses 1,2,3,4,11 with calculated H_2O content to reach observed cation sums and normalized on the basis of 138.23 O pfu.

Table S2. Standards used:

Standard	WDS line and analyzing crystal
$Ba_2NaNb_5O_{15}$	Ba*L*α, LLIF; Nb*L*α, LPET
$MnNb_2Ta_2O_9$	Ta*L*α, LLIF
titanite	Ti*K*α, LLIF
zircon	Zr*L*α, LPET
ThO_2	Th*M*α, LPET
diopside	Si*K*α, TAP; Ca*K*α, LPET
andalusite	Al*K*α, TAP
$LaPO_4$	La*L*α, LLIF
$CePO_4$	Ce*L*α, LLIF
$PrPO_4$	Pr*L*β, LLIF
$NdPO_4$	Nd*L*β, LLIF
$SmPO_4$	Sm*L*β, LLIF
$DyPO_4$	Dy*L*β, LLIF
YPO_4	Y*L*α, LPET
fayalite	Fe*K*α, LLIF
spessartine	Mn*K*α, LLIF
forsterite	Mg*K*α, LTAP
orthoclase	K*K*α, LPET
albite	Na*K*α, TAP
pollucite	Cs*L*α. LPET
F-bearing riebeckite	F*K*α, LTAP *

*Overlap of Ba*L*β on Ce*L*α and Ce*Mζ* on F*K*α were accounted for; elements Zn, Sr, Gd, and U were sought but not detected.

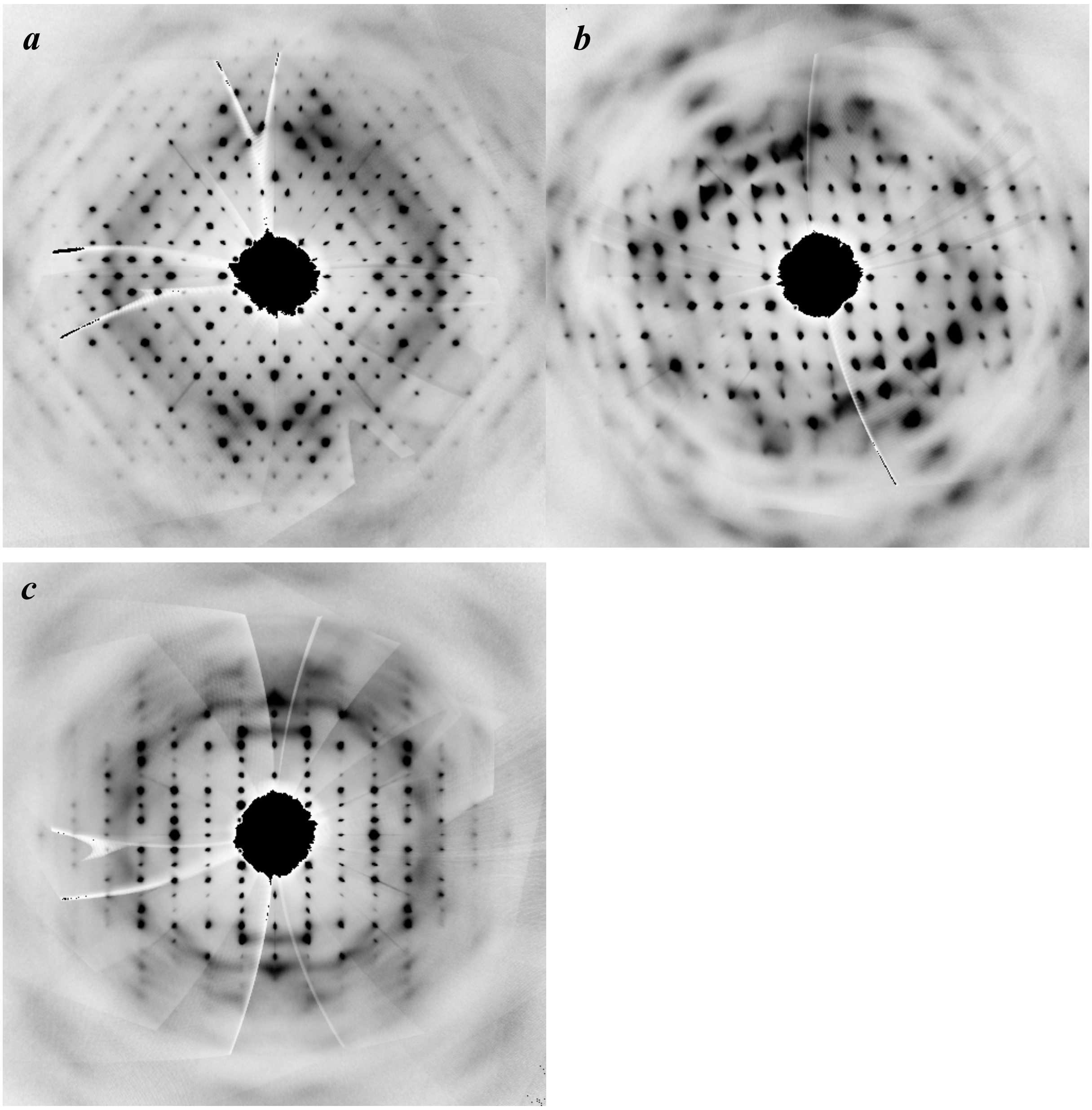

Figure S1 Unwrapped sections of the reciprocal space .a *hk*0, b *h*0*l*, c 0*kl* for crystal ILM02.
Note that the mosaicity starts at lower resolution along [-101], the direction of the ribbons in the (010) layers.

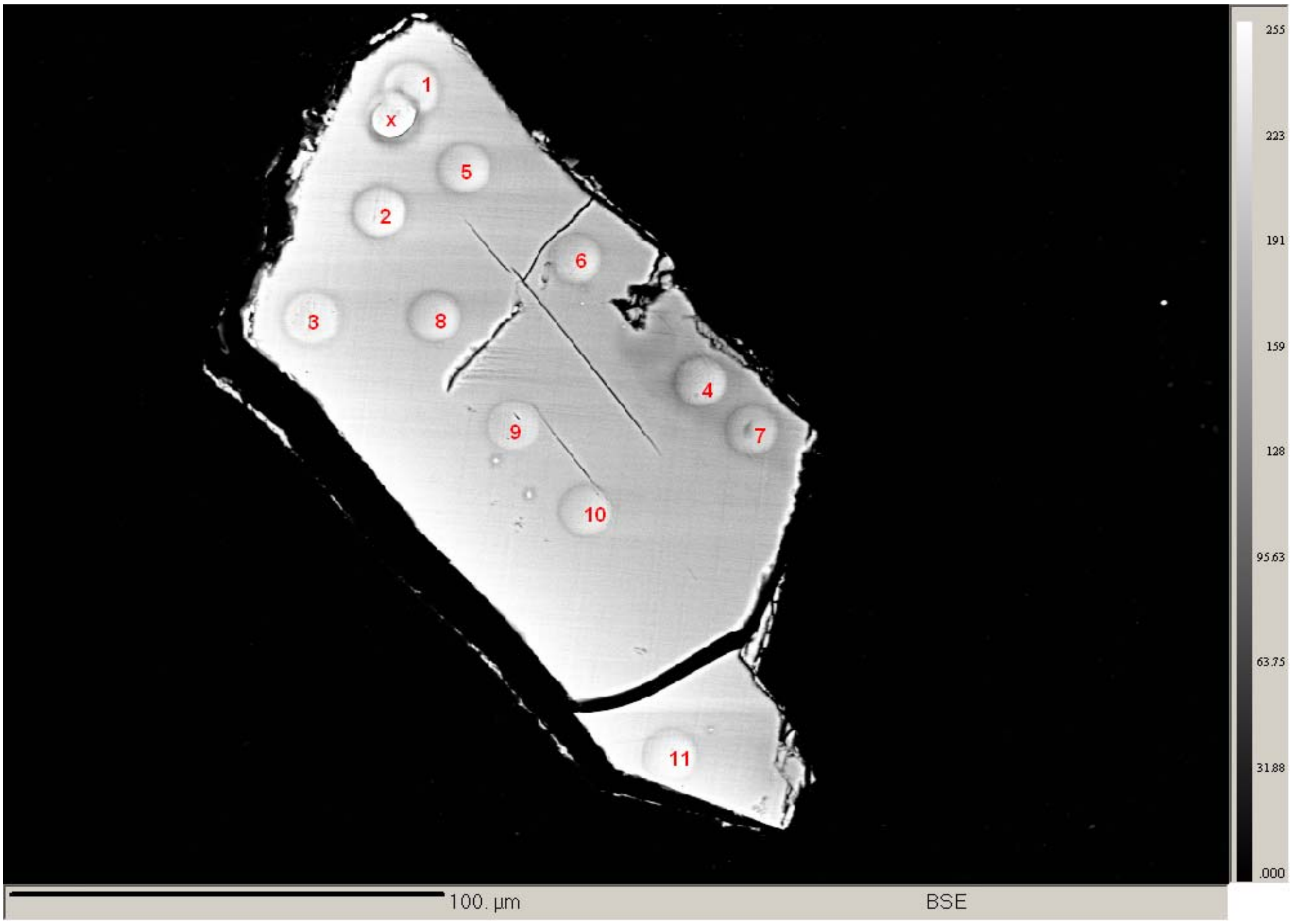

Figure S2. BSE image of the analyzed crystal. Numbers correspond to analyses in Table 3. X is a test spot to check beam sensitivity at 10nA and 15 µm Ø (i.e. counts vs. time test), no quantitative data acquired. The damage of the beam is evident.
